# Influence of talent retention strategy on employees׳ attitude to work: Analysis of survey data

**DOI:** 10.1016/j.dib.2018.08.103

**Published:** 2018-08-30

**Authors:** Olumuyiwa A. Oludayo, Comfort O. Akanbi, Bridget M. Obot, Segun I. Popoola, Aderemi A. Atayero

**Affiliations:** a Department of Business Management, Covenant University, Ota, Nigeria; b Department of Electrical and Information Engineering, Covenant University, Ota, Nigeria; c IoT-Enabled Smart and Connected Communities (SmartCU) Research Cluster, Covenant University, Ota, Nigeria

**Keywords:** Talent Retention, Employee, Attitude to work, Organizations

## Abstract

In this data article, an analysis on the strategies for talent retention in Covenant University and the corresponding effects on employees’ attitude to work was presented. The study population included the academic staff of Covenant University, which has a population of 530 employees, but a sample size was determined using Yamen׳s formula. The data obtained through survey questionnaires were analysed using Statistical Package for Social Sciences (SPSS). Linear regression was used to model the effect of talent retention strategy on employees’ attitude to work. This information is made publicly available to aid empirical researches on the subject of talent management in organizations.

**Specifications Table**

**Table:** 

Subject area	*Business Management*
More specific subject area	*Human Resource Management*
Type of data	*Table*
How data was acquired	*The data was collected majorly through the distribution of copies of questionnaires to covenant university academic staff*
Data format	*Raw, analyzed, inferential statistical data*
Experimental factors	*Sample consisted of academic staff of Covenant University. The researcher-made questionnaire which contained data on talent retention strategies and employee attitude to work were completed.*
Experimental features	*Descriptive data, ANOVA and Regression analysis of the data are represented using tables.*
Data source location	*Covenant University, Canaanland, Ota, Nigeria (Latitude 6.6718*^*o*^*N, Longitude 3.1581*^*o*^*E)*
Data accessibility	*In order to encourage evidence-based research in talent management strategies in organizations, detailed dataset are made publicly available*
Related research article	*Nil*

**Value of the data**

• The data provided in this article will make it easier to explore the issue of talent retention strategies and its consequential effect on employees’ attitude to work.
• The provision of this dataset will enable the management of organizations to see ways of improving talent retention strategies to enable employees perform better. It makes room for the achievement of the organizations goal because when the employees are happy, the customers are also happy.
• Further analysis of this dataset would enhance the skills, abilities and provides solution to employees’ level and satisfaction, build positive attitudes and increase productivity level.
• Further exploration of these data by the global professional research bodies will enhance the use of effective talent retention strategies to be applied in organizations across countries.
• Data shared in this data article will open up doors for new research collaborations.

## 1 Data

Talent management is a global challenge but the problem appear to be more acute for business organizations in developing countries as the demand for talented employees in this region keeps rising against its supply [1]. A global view of talent management in human resource development was provided in [2] to ensure balanced understanding. Nankervis [3] has identified the need to attract and retain required talent as a potential approach to sustaining the present economic boom experienced in China. Björkman and Smale [4] discussed some practical solutions that will help in handling the challenges associated with talent identification and development in multinational companies. Tafti et al. [5] attributed the problems and successes of talent management in Iranian automotive industries to structural, environmental, behavioural, and managerial factors. The consequences and prospects of proper handling of global talent challenges were presented in [6] with respect to international human resource management. Some organizations in India have adopted social networking as a veritable strategy for organizational branding and talent retention [7]. Mensah and Bolawole [8] presented a more compelling explanation concerning the positive relationship between talent management and employees’ work attitude using person-organization fit.

Most organizations in developing countries are now seeking viable means of retaining their workers by making their organizations more attractive and by training and developing the capacity of the limited human resources. Multinational companies are developing global systems that helps in identifying and developing talent [4]. However, most firms are still faced with the challenge of getting the required number of competent workers for the right positions and at the appropriate time [1]. Therefore, it is necessary to develop an effective talent management strategies that will positively influence employees’ attitude to work towards the ultimate purpose of talent retention within an organization.

## 2 Experimental design, materials, and methods

In this data article, the effects of the talent retention strategies adopted at Covenant University, Ota, Nigeria are measured with respect to employees’ attitude to work. An appropriate survey questionnaire was designed and administered to a total sample size of 200 respondents. The rate of response to survey questionnaire is presented in Table 1. 152, representing 76% of the total distributed questionnaire, were returned while 48, representing 24% of the total distributed questionnaire, were not returned.

**Table 1:** Rate of response to survey questionnaire.

**Questionnaire**	**Respondents**	**Percentage of respondents**
Returned	152	76
Not returned	48	24
Total	228	100

Table 2 shows the demography of 152 respondents which comprises of gender, age, and educational attainment. The gender distribution showed that 71 respondents (46.7%) were male while the remaining 81 respondents (53.3%) were female. This implies that the female respondents had the highest percentage. The age distribution showed that 40 (26.3%) of the respondents were within the age range of 20 and 30 years. 39 (25.7%) of the respondents were between 31 and 40 years old. 57 (37.5%) of the respondents were between 41 and 50 years old, while the remaining 16 (10.5%) were 51 years old and above. This implies that a greater number of the respondents were averagely between the age of 41 and 50. The educational attainment was categorized into three – Higher National Diploma (HND)/Bachelor of Science (B.Sc)/Bachelor of Art (B.A), Master of Science (M.Sc)/Master of Business Administration (MBA)/Master of Art (M.A), and Doctorate degree (Ph.D). 42 (27.6%) of the respondents were first degree holders. 54 (35.5%) had Masters’ degrees while the remaining 56 (36.8%) had doctorate degrees.

**Table 2:** Demographic data of the respondents.

**Social characteristics**		**Percentage (%)**
Gender	Male	46.7
	Female	53.3
Age	21–30 years	26.3
	31–40 years	25.7
	41–50 years	37.5
	51 years and above	10.5
Educational attainment	HND/B.Sc/B.A	27.6
	M.Sc/MBA/M.A	35.5
	Ph.D	36.8

Responses to the survey questionnaires were analysed based on operationalization of variables into dependent and independent variables. These variables were analysed with different sets of questions listed in the questionnaire. The first set of analyses was conducted on the independent variable i.e. talent retention. The second set of analyses was conducted on the dependent variable i.e. employees’ attitude to work.

The responses of the respondents to different questions on talent retention strategies are presented in Table 3, Table 4, Table 5, Table 6, Table 7. Table 3 presents the summary of the responses to the question asked on whether the employee agreed that compensations were paid to workers in the case of any form of accident sustained on job. 2 (1.3%) respondents indicated that they disagreed with the notion. 2 (1.3%) respondents were undecided about the question. 66 (43.4%) respondents agreed that compensation paid to workers in the case of any form of accident sustained on job is satisfactory while 82 (53.9%) strongly agreed to the same notion.

**Table 3:** Data on responses to whether compensation paid to workers in the case of any form of accident sustained on job is satisfactory.

		**Frequency**	**Percent**	**Valid percent**	**Cumulative percent**
Valid	Disagree	2	1.3	1.3	1.3
	Undecided	2	1.3	1.3	2.6
	Agree	66	43.4	43.4	46.1
	Strongly agree	82	53.9	53.9	100.0
	Total	152	100.0	100.0

**Table 4:** Data on responses to whether the organization provides opportunities for professional growth.

		**Frequency**	**Percent**	**Valid percent**	**Cumulative percent**
Valid	Disagree	0	0.0	0.0	0.0
	Undecided	1	0.7	0.7	0.7
	Agree	69	45.4	45.4	46.1
	Strongly agree	82	53.9	53.9	100.0
	Total	152	100.0	100.0

**Table 5:** Data on the responses to whether the organization׳s policies for promotion and advancement are fair.

		**Frequency**	**Percent**	**Valid percent**	**Cumulative percent**
Valid	Disagree	0	0.0	0.0	0.0
	Undecided	4	2.6	2.6	2.6
	Agree	50	32.9	32.9	35.5
	Strongly agree	98	64.5	64.5	100.0
	Total	152	100.0	100.0

**Table 6:** Data on the responses to whether talent is valued in this organization.

		**Frequency**	**Percent**	**Valid percent**	**Cumulative percent**
Valid	Disagree	0	0.0	0.0	0.0
	Undecided	1	0.7	0.7	0.7
	Agree	72	47.4	47.4	48.0
	Strongly agree	79	52.0	52.0	100.0
	Total	152	100.0	100.0

**Table 7:** Data on the responses to whether the training programs have improved overall performance.

		**Frequency**	**Percent**	**Valid percent**	**Cumulative percent**
Valid	Disagree	0	0.0	0.0	0.0
	Undecided	1	0.7	0.7	0.7
	Agree	71	46.7	46.7	47.4
	Strongly agree	80	52.6	52.6	100.0
	Total	152	100.0	100.0	

Table 4 presents the responses to the research statement “The organization provides opportunities for professional growth”. Only 1 respondent was undecided about the statement. 69 (45.4%) respondents agreed while 82 (53.9%) respondents strongly agreed to the statement.

Table 5 presents the summary of the data on the responses to the research statement “This organization׳s policies for promotion and advancement are fair.” 4 respondents are undecided while 98 respondents strongly agree to the notion, however, 50 (32.9%) respondents agreed to the notion.

Table 6 presents the summary of data on the responses to the research statement “Talent is valued in this organization”. One respondent was undecided, 72 respondents agreed, while 79 strongly agreed to the notion.

Table 7 presents the summary of data on the responses to the research statement “The training programs have empowered me in my overall performance”. The data shows that one (0.7%) respondent was undecided, 71 (46.7%) indicated agreement to the notion, while 80 (52.6%) respondents strongly agreed.

Table 8, Table 9, Table 10, Table 11 presents statements that relate to employees attitude to work. These statements includes: (1) I get a feeling of personal satisfaction from my work; (2) I feel a strong commitment to the goals and values of this organization; (3) I display good organizational citizenship behaviour and; (4) I receive encouragement that gives me a sense of belonging.

**Table 8:** Data on feeling of personal satisfaction from work.

		**Frequency**	**Percent**	**Valid percent**	**Cumulative percent**
Valid	Disagree	0	0.0	0.0	0.0
	Undecided	1	0.7	0.7	0.7
	Agree	70	46.1	46.1	46.7
	Strongly agree	81	53.3	53.3	100.0
	Total	152	100.0	100.0

**Table 9:** Data on the feeling of a strong commitment to the goals and values of the organization.

		**Frequency**	**Percent**	**Valid percent**	**Cumulative percent**
Valid	Disagree	0	0.0	0.0	0.0
	Undecided	1	0.7	0.7	0.7
	Agree	85	55.9	55.9	56.6
	Strongly agree	66	43.4	43.4	100.0
	Total	152	100.0	100.0

**Table 10:** Data on the display good organizational citizenship behaviour.

		**Frequency**	**Percent**	**Valid percent**	**Cumulative percent**
Valid	Undecided	0	0.0	0.0	0.0
	Disagree	1	0.7	0.7	0.7
	Agree	76	50.0	50.0	50.7
	Strongly agree	75	49.3	49.3	100.0
	Total	152	100.0	100.0

**Table 11:** Data on whether the respondent received encouragement that gives a sense of belonging.

		**Frequency**	**Percent**	**Valid percent**	**Cumulative percent**
Valid	Undecided	0	0.0	0.0	0.0
	Disagree	1	0.7	0.7	0.7
	Agree	80	52.6	52.6	53.3
	Strongly agree	71	46.7	46.7	100.0
	Total	152	100.0	100.0	

Regression analyses were performed on the inferential statistical data obtained. The adoption of this test statistics is based on the fact that regression analysis is used in describing the dependence of variable on one or more variables. It is also used to determine the effect and the impact of the independent variable on the dependent variable.

Therefore, from this equation, employees’ attitude to work is dependent on talent retention strategies. This is expressed as:

EA=f(TR)

Where, EA = *Y*TR = *X*

$$\;\;\;\;Y=C+\beta_{1}X+µ$$

Where *X*,*Y* are defined above

*C*= Constant

*β*_1_= Co-efficient of X

*µ* = Error term

Model summary, results of the Analysis of Variance (ANOVA), and the coefficients of the independent variables are presented in Table 12, Table 13, Table 14 respectively.

**Table 12:** Model summary.

**Model**	***R***	**R squared**	**Adjusted R squared**	**Standard error of the estimate**
1	0.410	0.168	0.162	0.33368

**Table 13:** Analysis of variance (ANOVA) result.

**Model**	**Sum of squares**	**df**	**Mean square**	**F**	**Sig.**
Regression	3.368	1	3.368	30.247	0.000
Residual	16.701	150	0.111		
Total	20.069	151

**Table 14:** Coefficients of the variables.

**Model**	**Unstandardized coefficients**		**Standardized coefficients**	***t***	**Sig.**
	B	Standard error	Beta		
Constant	2.581	0.345		7.486	0.000
Talent retention	0.417	0.076	0.410	5.500	0.000

## 3 Ethical considerations

In this study, the goals of the research were plainly made known to all participants to encourage active and objective participation. In order to uphold the ethical values during data collection, respondents were allowed to remain anonymous and all personal information obtained were treated as confidential.

## 4 Conclusion

This data article presented and analyzed the statistical information on talent retention strategies in organizations and what effect it has on employee׳s attitude to work with a case study of Covenant University, Nigeria. The dataset provided in this article will encourage further quantitative analysis into the subject matter. Free accessibility to these data will give management, executives and human resource consultants’ useful information for better decision making in the management of human resources.
